# Novel Anticoagulants in Atrial Fibrillation: Monitoring, Reversal and Perioperative Management

**DOI:** 10.1155/2015/424031

**Published:** 2015-07-02

**Authors:** Fadi Shamoun, Hiba Obeid, Harish Ramakrishna

**Affiliations:** ^1^Division of Cardiovascular Diseases, Mayo Clinic, Phoenix, AZ, USA; ^2^Damascus University Faculty of Medicine, Damascus, Syria; ^3^Division of Cardiovascular and Thoracic Anesthesiology, Mayo Clinic, Phoenix, AZ 85054, USA

## Abstract

Atrial fibrillation continues to be a significant source of morbidity and mortality worldwide. Effective anticoagulation remains the cornerstone of outpatient and inpatient treatment. The use of the new generation of anticoagulants (NOACs) continues to grow. Recently published data indicate their cost-effectiveness and overall safety in stroke prevention; compared to vitamin K antagonists, they can be prescribed in fixed doses for long-term therapy without the need for coagulation monitoring. Both United States and European Guidelines recommend NOACs for stroke prevention in patients with atrial fibrillation. This review discusses each of the NOACs, along with their efficacy and safety data. It explores the most recent guidelines regarding their perioperative use in atrial fibrillation patients. It also discusses bleeding complications, perioperative management, and reversal agents.

## 1. Introduction

Atrial fibrillation (AF) is one of the most common tachyarrhythmias in clinical practice. It accounts for about 35% of hospital admissions from cardiac arrhythmias. AF prevalence is projected to increase from 5.2 million in 2010 to 12.1 million cases in 2030 [1]. AF increases the risk of stroke 4-5-fold, independent of other cardiac or noncardiac morbidities [2]. At least 15–20% of all ischemic strokes are due to AF. Also, AF is an independent risk factor for stroke recurrence [3]. Penado et al. showed that the hazard ratio for recurrent stroke among those with AF who were not treated with anticoagulants was 2.1 (95% confidence interval (CI): 1.4 to 2.9; *P* < 0.001), whereas the hazard ratio for recurrent severe stroke was 2.4 (95% CI: 1.6 to 3.6; *P* < 0.001) [3].

## 2. Warfarin

Warfarin has been the most common medication used for anticoagulation [4]. It has established its effectiveness in preventing thromboembolic events in patients with AF. At least 1% of the population in the United Kingdom is taking warfarin, as well as 8% of those aged over 80 years.

Warfarin use is associated with many undesired side effects that could significantly affect patients' well-being. The challenges associated with warfarin therapy often outweigh its benefits [5].

A study by Birman-Deych et al. shows that about one-third of AF patients who are ideal candidates for warfarin therapy are not offered the treatment [6]. That is especially true for the black and Hispanic population. Another study by Hylek et al. published in 2007 shows that 26% of patients 80 years of age or older stop taking warfarin within 1 year of treatment despite ongoing indication [7]; 81% of those patients stopped warfarin due to safety concerns.

A study to assess the prevalence of hospital admissions due to adverse drug reactions in the adult population concluded that warfarin is the leading drug causing these hospitalizations with a rate of 33.3% of all admissions due to adverse drug events [8].

Of all types of bleeding associated with warfarin therapy, intracranial hemorrhage (ICH) is the most significant [9]. ICH is mainly responsible for the majority of deaths and disabilities caused by warfarin-related bleeding.

Monitoring of warfarin is easily achievable by testing prothrombin time (PT) and measuring the international normalized ratio (INR) values. The target INR that is required in AF patients is between 2 and 3. Home monitoring of INR has proven to reduce the risk of thromboembolism, bleeding event, and death [10].

The major side effect of warfarin is bleeding; the risk of bleeding increases when the INR is higher than 3. When INR is within therapeutic range and an elective surgery is needed, warfarin should be stopped for at least 5 days. For patients who are bleeding or require rapid reversal of warfarin due to serious bleeding or emergency surgery, vitamin K should be given at 10 mg with prothrombin complex concentrate (PCC) (25–50 IU/kg) or fresh frozen plasma (15–30 mL/kg) [11].

## 3. Aspirin versus Warfarin

Since the risk of bleeding increases with age, some have suggested that using aspirin in elderly patients could be a suitable alternative to warfarin; however, the Birmingham Atrial Fibrillation Treatment of the Aged (BAFTA) study confirmed that aspirin was associated with the same rate of bleeding events (1.9% versus 2.0% risks per year; 0.97 relative risk (RR), 95% CI: 0.53–1.75), and worse primary outcomes, ICH, arterial embolism, or stroke (yearly risk 1.8% in the warfarin group versus 3.8% in the aspirin group, RR 0.48, 95% CI: 0.28–0.80, *P* = 0.003) [12].

## 4. Novel Oral Anticoagulants (NOACs)

The novel oral anticoagulants (NOACs) appear to be a good alternative to traditional anticoagulation with vitamin K antagonists (VKAs). They have better oral bioavailability with less food and drug interactions. They do not require frequent INR monitoring and seem to be well tolerated in the long-term use. A systematic review and meta-analysis of 5 phase 3 clinical trials that studied the efficacy and safety of NOACs was published in 2012 [13]. It compared warfarin to NOACs in AF patients. NOAC use was associated with decreased stroke and systemic embolism (RR: 0.82; 95% CI: 0.69–0.98; *P* = 0.03) as well as all-cause mortality (RR: 0.91; 95% CI: 0.85–0.96; *P* = 0.0026) compared with warfarin. The analysis showed better safety outcomes associated with NOACs; the RR of major bleeding was 0.83 (95% CI: 0.69–1.002; *P* = 0.055). Also, the RR of hemorrhagic stroke was significantly low (RR: 0.51; 95% CI: 0.41–0.64; *P* ≤ 0.001).

The 4 medications that are currently available in the market are dabigatran (a direct thrombin inhibitor), rivaroxaban, edoxaban, and apixaban (factor Xa inhibitors). All of these agents (except edoxaban) are approved in the United States, the European Union, and Canada for the indication of nonvalvular AF. Currently, edoxaban is under evaluation. Rivaroxaban is the most commonly prescribed NOAC in the United States.

There are still many questions about the NOACs that require more randomized data relating to perioperative use, particularly relating to cessation of anticoagulant therapy in surgical patients who need emergent procedures.

A systematic review by Harel et al. examined the efficacy and safety of NOACs compared to VKAs by studying the data from 8 randomized controlled trials that included patients with AF or venous thromboembolism, and associated chronic kidney disease (creatinine clearance (CrCl) = 30–50 mL/min) [14]. It is concluded that there was no major difference in the primary efficacy outcomes or the primary safety outcomes with NOACs compared to VKAs; however, dose adjustments in renal failure as well as choice of optimal NOAC in this high-risk group remain important clinical questions. Several questions have been posed about NOAC use in elderly patients. There has been a recently published meta-analysis on the safety of newer anticoagulants in elderly patients [15]. This is an important point given the fact that the patients included in the recent trials were a relatively younger group of patients.

The combination of antiplatelet agents, specifically the use of dual antiplatelet agents, remains an important question. At this point, there are no clear guidelines to help understand the risk of bleeding in patients who require the combination of antiplatelets and anticoagulants.

Finally, it is important to note that with the newer anticoagulants, we are seeing different types of bleeding with less retroperitoneal and ICHs compared to warfarin. That is believed to be due to abundance of factor VII on the blood-brain barrier that is affected by warfarin and not the newer anticoagulants [16].

## 5. Dabigatran

Dabigatran is the only direct thrombin inhibitor currently approved for stroke prevention in patients with nonvalvular AF. The doses approved in the United States are 150 mg or 75 mg twice daily; 80% is renally cleared [17]. The 75 mg dosage is indicated when the patients have poor renal function (CrCl 15–30 mL/min) or are on P-glycoprotein inhibitors with poor CrCL 30–50 mL/min. Dabigatran is contraindicated when CrCl is <15 mL/min.

The RE-LY trial compared warfarin and dabigatran for the prevention of stroke and systemic embolism in AF patients [18]. Patients were divided into 3 groups. The first group received warfarin; the second received dabigatran 110 mg; and the third received dabigatran 150 mg. Low-dose dabigatran was found to be noninferior to warfarin (RR 0.91; 95% CI: 0.74 to 1.11; *P* < 0.001). High-dose dabigatran was superior to warfarin (RR 0.66; 95% CI: 0.53 to 0.82; *P* < 0.001). Major bleeding was lower in the 110 mg group (*P* = 0.003) and similar to warfarin in the 150 mg group (*P* = 0.31).

### 5.1. Dabigatran Monitoring

Although there is no need to monitor dabigatran routinely when given to AF patients, there are certain clinical settings when monitoring becomes a necessity [19], such as in the setting of urgent surgery, where elevated dabigatran plasma levels can raise the risk of bleeding. Supratherapeutic levels in patients who are experiencing adverse effects due to decreased clearance of dabigatran (possibly because of deteriorating renal function) are a real concern.

Dabigatran prolongs the activated partial thromboplastin time (aPTT) more than the PT (it affects the intrinsic coagulation pathway more than the extrinsic coagulation pathway) [20]. A recent study comparing different tests for monitoring dabigatran levels in patients with AF found a strong correlation between the total and free dabigatran plasma levels measured by liquid chromatography-tandem mass-spectrometry (LC-MS/MS) and indirect measurements by Hemoclot Thrombin Inhibitor (HTI) and ecarin clotting time (ECT) assays (*P* < 0.001) [21]. This correlation suggests that HTI and ECT assays are highly sensitive for the assessment of dabigatran activity when compared to standard coagulation tests (aPTT, PT).

In another study by Hapgood et al., investigators measured the dabigatran concentrations by the Hemoclot assay and correlated the results with aPTT and thrombin time (TT) [22]. They found that TT was very sensitive to the presence of the drug and that aPTT is useful as a qualitative test (to determine whether dabigatran is having an anticoagulant effect in the patient), but both TT and aPTT had only moderate correlation with the drug levels. This could be useful in preparing patients for surgery in settings where HTI or ECT assays are not available. The study recommended measuring aPTT and TT before elective surgery in patients taking dabigatran.

### 5.2. Dabigatran Reversal

The half-life of dabigatran is between 12 and 14 hours [20] and up to 18 hours when the CrCl drops to 30–50 mL/min and 27 hours when the CrCl is less than 30 mL/min. In patients with normal renal function, the steady-state trough level should be diminished by 75% after discontinuation of dabigatran for 24 hours. Therefore, stopping dabigatran administration is simply enough for most of the cases that require reversal of its effect. Patients in the perioperative period are recommended to stop dabigatran at least 24 hours prior to low-risk surgery if kidney function is normal, and at least 48 hours before surgeries with a high risk of bleeding [23]. If the CrCl is 31 to 50 mL/min, the last dose should be at least 48 hours before the procedure for low-risk surgery and 4 days before a procedure that poses a high risk of bleeding [20]. If the CrCl is less than 30 mL/min, dabigatran should be permanently discontinued, and any surgical intervention should be deferred for at least 5 days.

In the case of an emergent surgical procedure or severe bleeding, stopping the drug may not be sufficient. Limited data and studies are available to identify the best reversal method. Transfusion of packed red blood cells, fresh frozen plasma, and surgical interventions to stop the bleeding are suggested as a supportive therapy. Administration of activated charcoal could be useful to inhibit the absorption of dabigatran from the gastrointestinal tract if a recent ingestion has been reported. Dabigatran can also be dialyzed in patients with renal impairment. A study that enrolled 23 patients with different stages of renal impairment investigated the fraction of dabigatran that could be eliminated from the blood after hemodialysis [24]. It concluded that hemodialysis removed 62% of dabigatran after 2 hours and 68% after 4 hours.

**Figure 1:**
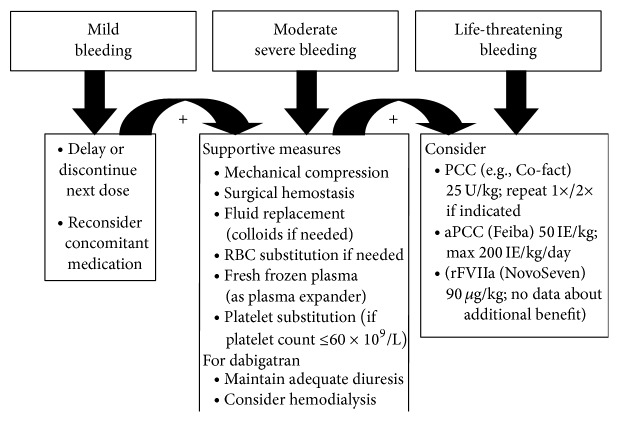
Recommendation for bleeding treatment while on NOACs.

Nonspecific therapies (activated factor VIIa or PCC) can also be considered. A randomized controlled study by Eerenberg et al. compared the effect of nonactivated PCC versus saline to reverse the anticoagulation of either dabigatran or rivaroxaban in 12 healthy subjects [25]. In this trial, dabigatran was administered at a dose of 150 mg, and it increased aPTT, ECT, and TT. This was followed by administration of a single bolus of 50 IU/kg PCC; the PCC failed to restore these coagulation tests to their normal value. The study concluded that PCC is not effective as an antidote for dabigatran; however, Bernstein et al. have noticed that the PCC administered in the previous study was not activated and proposed an activated form of PCC as an alternative to reverse the dabigatran effect [26]. This proposal was made based on a trial by van Ryn et al. [27], which concluded that FEIBA (which is an activated PCC) reversed the prolonged bleeding time in rats treated with dabigatran (Figure 1).

In May 2013, Schiele et al. reported for the first time a specific antidote for dabigatran [28]. They generated an antibody fragment (aDabi-Fab (idarucizumab)) that could bind to the dabigatran molecule and reverse its effect in vitro and in vivo. aDabi-Fab mimics the thrombin molecule and is able to bind to dabigatran with an affinity that is 350 times more than the affinity of dabigatran to thrombin, but it has no functional thrombin mimicking activity, and it does not induce coagulation. Schiele et al. infused rats with dabigatran until they reached a 4-fold prolongation of TT and 2-fold prolongation of aPTT. They found that a single bolus injection of aDabi-Fab was able to restore TT and aPTT to normal within 1 minute. In April 2014, another trial on pigs showed that aDabi-Fab was able to reverse the effect of dabigatran even when it was given in supratherapeutic levels and when severe bleeding was induced by trauma [29].

Van Ryn et al. presented a study on 35 healthy volunteers which showed that dabigatran inhibited the fibrin formation after a small scratch, and idarucizumab was able to completely reverse this effect and restored fibrin formation [30]. Idarucizumab is currently investigated in real life bleeding events in patients who are receiving dabigatran. This study (RE-VERSE AD) is going to take place in 35 different countries including the United States.

PER977 is another synthetic small molecule under development that has shown to reverse dabigatran as well as other NOACs ex vivo in human blood and decreased bleeding in a standard rat tail bleeding model [31]. This new antidote is currently undergoing more clinical trials.

## 6. Rivaroxaban

Rivaroxaban is the first direct factor Xa inhibitor. It is dosed once daily; 40% is excreted through the kidney and the remaining one-third is metabolized in the liver and excreted in the feces. The recommended dose is 20 mg once a day for patients with CrCl >50 mL/min and 15 mg once daily for those with CrCl <50 mL/min. Rivaroxaban was noninferior to warfarin in the prevention of stroke and systemic embolism in ROCKET AF [32, 33], but it had better outcomes in terms of life-threatening bleeding events (ICH and fatal bleeding).

Even though the hepatic metabolism of rivaroxaban could help eliminate the drug in cases of renal failure, further studies should be conducted to make sure it is effective and safe in this patient population. Thus, rivaroxaban is contraindicated in patients with CrCl <15 mL/min for the treatment of AF. It is also contraindicated in deep venous thrombosis and pulmonary embolism prophylaxis when with the CrCl <30 mL/min. Its use should be avoided in patients with moderate or severe hepatic impairment (Child-Pugh B and Child-Pugh C, resp.) [34].

### 6.1. Rivaroxaban Monitoring

As with dabigatran, rivaroxaban does not require monitoring except in certain circumstances. The PT has a linear correlation with rivaroxaban concentrations in the plasma [35]; however, PT results may vary with different reagents. For example, when using Neoplastin Plus (a thromboplastin reagent), PT doubles when rivaroxaban concentration is 301 *μ*g/L. When using Innovin (a different reagent), PT doubles when rivaroxaban concentration is 700 *μ*g/L. This result variation is mainly caused by different sensitivities of these reagents to rivaroxaban. This variation cannot be fixed by conversion of PT to INR; therefore, INR should not be used to evaluate rivaroxaban activity. A normal PT indicates no rivaroxaban activity [35].

Samama et al. proposed that anti-Factor Xa chromogenic assays are the best way for the estimation of rivaroxaban concentrations (when using standard calibration curves generated with the use of rivaroxaban calibrators and controls) [35]; however, there are 2 points that should be considered when using the chromogenic assays. The first one is that the assay measures the drug concentration in the plasma, not its activity, which means that a high level in the plasma does not necessarily indicate higher activity and, therefore, a higher risk of bleeding. The second point is that the results will be different depending on the time of blood sampling after rivaroxaban administration. For example, the plasma level of rivaroxaban will be higher after 2–4 hours of intake when compared to after 12 hours of intake. This should be considered when evaluating the treatment with rivaroxaban.

### 6.2. Rivaroxaban Reversal

Rivaroxaban has a half-life of 7–11 hours in patients with normal kidney function [24]. If an elective surgery is planned, rivaroxaban should be stopped for at least 24 hours before low-risk surgery or 48 hours before high-risk surgery. It can be resumed after 6–10 hours if the patient has normal kidney function (CrCl > 30 mL/min) and hemostasis has been achieved. If CrCl is below 30 mL/min, it should be stopped 2 days prior to low-risk surgery and 4 days prior to high-risk surgery. It is important to note that, unlike dabigatran, rivaroxaban cannot be dialyzed due to the high plasma protein binding capacity of this medication (95% is bound to plasma proteins).

The previously mentioned study by Bernstein et al. that evaluated Co-fact© (a nonactivated PCC) for the reversal of anticoagulation showed that rivaroxaban effect (prolongation of PT) was immediately and completely reversed by PCC (PT came back to normal) [26]. The endogenous thrombin potential was inhibited by rivaroxaban and normalized after PCC bolus as well. Since this study was performed on healthy individuals, more studies should be done to confirm the benefit of PCC in real-life bleeding situations.

The recombinant protein, PRT064445, was suggested by Lu et al. as a specific antidote for all direct and indirect factor Xa inhibitors [36]. It has the ability to reverse the effect of rivaroxaban in rabbits, by binding to the free factor Xa inhibitor concentration in plasma, and, therefore, decreasing its activity. It also succeeded in the management of blood loss induced in rats after administration of enoxaparin and fondaparinux.

A recent phase 2 study was designed to test another agent, andexanet alfa (PRT4445) [37]. The study reported that this antidote was able to dose-dependently reverse the effect of rivaroxaban in healthy volunteers. Also, it was well tolerated and did not cause any significant adverse effects. Andexanet alfa is now being studied in a phase 3 clinical trial known as ANNEXA-R to evaluate its efficacy and safety in reversing rivaroxaban [38].

## 7. Apixaban

Apixaban is the second direct factor Xa inhibitor. It is dosed twice daily and mainly excreted through the liver. The dose is 5 mg twice daily and could be reduced to 2.5 mg twice daily if patients meet 2 of 3 criteria: age 80 years, body weight 60 kg, or serum creatinine level 1.5 mg/dL.

Apixaban is superior to aspirin in the phase 3 AVERROES clinical trial [39]. It reduced significantly the stroke and pulmonary embolism events (hazard ratio with apixaban, 0.45; 95% CI: 0.32 to 0.62; *P* < 0.001). The risk of major bleeding appeared to be similar compared to aspirin in that trial (hazard ratio with apixaban, 1.13; 95% CI: 0.74 to 1.75; *P* = 0.57). Another phase 3 clinical trial, ARISTOTLE, showed apixaban to be superior to warfarin in prevention of stroke and systemic embolism in patients with AF (hazard ratio with apixaban, 0.79; 95% CI: 0.66 to 0.95; *P* < 0.001 for noninferiority; *P* = 0.01 for superiority) [40]. The risk of major bleeding was also lower in the apixaban group compared to the warfarin group (hazard ratio, 0.69; 95% CI: 0.60 to 0.80; *P* < 0.001).

Thus far, apixaban appears to be probably the safest option in case of chronic kidney disease [41]. Hohnloser et al. [42] evaluated the outcomes of the ARSISTOLE trial in relation to renal function. They concluded that apixaban reduced the rate of stroke, death, and major bleeding, when compared to warfarin, regardless of renal function. Patients with estimated glomerular filtration rate of ≤50 mL/min (as determined by the Cockcroft–Gault and Chronic Kidney Disease Epidemiology Collaboration (CKD-EPI) equations) seemed to have greater relative risk reduction in major bleeding with apixaban (hazard ratio 0.50 (95% confidence interval: 0.38–0.66), *P* = 0.005). There are still limited data that compare NOACs to each other in specific populations such as patients with renal failure. For now, what we know is that apixaban is certainly a very promising anticoagulation treatment for this population.

PT, INR, and aPTT tests are not ideal to monitor apixaban; however, a normal PT value indicated no activity of apixaban and can be useful when other tests are not available. Becker et al. proved that there is a strong linear correlation between apixaban plasma concentration and apixaban activity when measured using a standard laboratory chromogenic anti-Xa assay with either low molecular weight heparin or apixaban calibrators [43]. Hence, measurement of apixaban antifactor Xa activity using chromogenic laboratory assays appears to be the most accurate method.

Just like rivaroxaban, apixaban should be stopped for 24 hours at least before low-risk surgery or 48 hours before high-risk surgery when CrCl is >30 mL/min [24]. If CrCl is below 30 mL/min, it should be stopped 2 days prior to low-risk surgery and 4 days prior to high-risk surgery. Apixaban cannot be dialyzed due to the high plasma protein binding capacity of this medication (87% is bound to plasma proteins).

A recent phase 2 study was designed to test the new specific antidote for the factor Xa inhibitors, andexanet alfa (PRT4445) for apixaban reversal [44]. The study administered 5 mg of apixaban to 54 healthy volunteers for 6 days. Then, the volunteers were given intravenous andexanet. The effect of apixaban was reversed within 2 minutes after the administration of the new antidote, by decreasing the concentration of the unbound apixaban in plasma. Currently, a phase 3 clinical trial is ongoing to study the efficacy and safety of andexanet alfa to reverse apixaban effect [38].

## 8. Edoxaban

Edoxaban is the third direct factor Xa inhibitor. It is dosed once daily and excreted through the liver. Edoxaban is not yet approved by the food and drug administration for the management of AF. One phase 3 clinical trial was conducted to evaluate its efficacy and safety as well as the best dosing regimen [45]. The study had 3 groups: the first group received warfarin; the second one received edoxaban 30 mg once daily; and the third group received edoxaban 60 mg once daily. High-dose edoxaban was noninferior to warfarin in the prevention of stroke and systemic embolism (HR 0.79; 97.5% CI: 0.63 to 0.99; *P* < 0.001) but had a higher rate of major bleeding (HR 0.80; 95% CI: 0.71 to 0.91; *P* < 0.001). The low-dose edoxaban was noninferior to warfarin as well (HR 1.07; 97.5% CI: 0.87 to 1.31; *P* = 0.005) and had a lower rate of major bleeding (HR 0.47; 95% CI: 0.41 to 0.55; *P* < 0.001).

Similar to other factor Xa inhibitors, chromogenic antifactor Xa assays can be used to measure the plasma concentrations of edoxaban when drug-specific calibrators are available [46]. A trial that evaluated the use of the reversal agent PER977 was published in November 2014 [47]. The study was on 80 healthy volunteers who were randomized into 8 cohorts (each cohort was assigned to a different dose of PER977 ranging from 5 to 300 mg). Eight persons in each cohort received PER799 intravenously, alone and after administration of edoxaban, and 2 persons in each cohort received placebo. The trial used whole-blood clotting time to measure the anticoagulant effect of edoxaban and its reversal by PER977. Whole-blood clotting time shows low variability and high reproducibility and correlates well with edoxaban plasma concentrations. The effect of edoxaban was successfully reversed and the whole-blood clotting time was restored to values close to baseline in those who received 100–300 mg of PER977 within 10–30 minutes (Table 1).

## 9. Conclusion

Dabigatran, rivaroxaban, and apixaban are effective and safe alternative to warfarin for the prevention of stroke and systemic emboli in patient with paroxysmal or permanent atrial fibrillation. NOACs have a wide therapeutic range with reasonable safety margin.

Dabigatran activity can be monitored using HTI and ECT assays. The best tests to monitor factor Xa inhibitors are antifactor Xa chromogenic assays when standard calibrators are available.

Specific antidotes for direct thrombin inhibitors and Xa inhibitors are underway. Clinical studies are currently ongoing to evaluate some suggested antidotes.

Tables 1, 2, and 3 and Figure 1 summarize the key data useful in perioperative management, including NOAC dosages, reversal options, and therapeutic options in bleeding patients.

Dabigatran is reversed by the administration of activated factor VIIa or activated PCC. Hemodialysis is also effecive in life-threatening emergencies.

## Figures and Tables

**Table 1:** Recommendation for NOACs cessation before elective procedure.

	Dabigatran		Apixaban		Rivaroxaban	

	No important bleeding risk and/or adequate local hemostasis possible: perform at trough level (i.e., ≥12 hours or 24 hours after last intake)					

Creatinine clearance	Low risk	High risk	Low risk	High risk	Low risk	High risk

≥80 mL/min	≥24 hours	≥48 hours	≥24 hours	≥48 hours	≥24 hours	≥48 hours
50–80 mL/min	**≥36 hours**	**≥72 hours**	≥24 hours	≥48 hours	≥24 hours	≥48 hours
30–50 mL/min	**≥48 hours**	**≥96 hours**	≥24 hours	≥48 hours	≥24 hours	≥48 hours
15–30 mL/min	Not indicated	Not indicated	**≥36 hours**	**≥48 hours**	**≥36 hours**	**≥48 hours**
<15 mL/min	No official indication for use					

**Table 2:** Recommendations for monitoring and reversal of NOACs.

NOAC	Trial name	Most accurate monitoring tests	Qualitative monitoring tests	Reversal
Dabigatran	RE-LY	HTI ECT	TT aPTT	(i) Activated charcoal (if a recent ingestion has been reported) (ii) FEIBA (activated PCC) (iii) Hemodialysis (iv) aDabi-Fab [idarucizumab]^*^ (v) PER977^*^

Rivaroxaban	ROCKET AF	Antifactor Xa chromogenic assays	PT	(i) Recombinant activated factor VII (rFVIIa) (ii) Co-fact© (a nonactivated PCC) (iii) FEIBA (activated PCC) (iv) Andexanet alfa (PRT4445) (ANNEXA-R)^*^ (v) PER977^*^
Apixaban	AVERROES ARISTOTLE			
Edoxaban	ENGAGE AF-TIMI			

^*^Those reversal agents are still under evaluation.

**Table 3:** Dosage recommendations for NOACs and contraindications [17, 34, 48].

NOACs	Dosage for stroke prevention	Indications for a reduced dosage	Contraindications
Dabigatran	150 mg twice daily	(i) 75 mg twice daily for those with CrCl 15–30 mL/min (ii) 75 mg twice daily for those on P-gp inhibitors in with CrCl 30–50 mL/min	(i) Patients with CrCl < 15 mL/min (ii) Active pathological bleeding (iii) Mechanical prosthetic heart valve (iv) Patients on P-gp inducer rifampin (v) Patients on P-gp inhibitors with CrCl <30 mL/min

*Rivaroxaban *	20 mg once a day	15 mg once daily for those with CrCl 15–50 mL/min	(i) Severe renal impairment (CrCL <15 mL/min) (ii) Moderate or severe hepatic impairment (Child-Pugh B or Child-Pugh C) or with any degree of hepatic disease associated with coagulopathy (iii) Nursing women (iv) Active pathological bleeding (v) Coadministration of combined P-gp and strong CYP3A4 inhibitors and inducers

Apixaban	5 mg twice daily	(i) 2.5 mg twice daily if patients meet 2 of 3 criteria: age 80 years, body weight 60 kg, or serum creatinine level 1.5 mg/dL (ii) 2.5 mg twice daily if coadministration of strong dual inhibitors of CYP3A4 and P-gp	(i) Active pathological bleeding (ii) Pregnant and nursing women (iii) Coadministration of strong dual inducers of CYP3A4 and P-gp

## Abbreviations

AF: Atrial fibrillation
aPTT: Activated partial thromboplastin time
CI: Confidence interval
CrCl: Creatinine clearance
ECT: Ecarin clotting time
HTI: Hemoclot Thrombin Inhibitor
ICH: Intracranial hemorrhage
INR: International normalized ratio
NOACs: Novel oral anticoagulants
PCC: Prothrombin complex concentrate
PT: Prothrombin time
RR: Relative risk
TT: Thrombin time
VKA: Vitamin K antagonist.
